# Assessing the Genomic Landscape of *Salmonella enterica* Isolated From Cattle Faeces on a Nigerian Farm

**DOI:** 10.1002/mbo3.70129

**Published:** 2025-11-12

**Authors:** Adewale A. Adetoye, Ayorinde O. Afolayan, Olabisi C. Akinlabi, Stella E. Ekpo, Isaac O. Olatoye, Funmilola A. Ayeni

**Affiliations:** ^1^ Department of Pharmaceutical Microbiology, Faculty of Pharmacy University of Ibadan Ibadan Oyo Nigeria; ^2^ Institute for Infection Prevention and Control Medical Center—University of Freiburg Freiburg Germany; ^3^ Department of Medical Microbiology and Infectious Diseases University of Manitoba Winnipeg Manitoba Canada; ^4^ Department of Veterinary Public Health and Preventive Medicine, Faculty of Veterinary Medicine University of Ibadan Ibadan Oyo Nigeria; ^5^ Department of Environmental and Occupational Health, School of Public Health Indiana University Bloomington Indiana USA

**Keywords:** antibiotics, antimicrobial resistance, cattle faeces, *Salmonella enterica*, susceptibility

## Abstract

Antibiotic resistance is a global menace, particularly in low‐ and middle‐income countries where antimicrobial resistance (AMR) in zoonotic pathogens like *Salmonella* is on the rise. This study investigates the phenotypic and genotypic AMR in *Salmonella enterica*. isolated from cattle faeces collected by faecal grab method on a Nigerian dairy farm. *Salmonella enterica* was cultured from the faecal samples of 138 individual cattle at the University of Ibadan dairy farm, with identification done through MALDI‐TOF‐MS, genus‐specific PCR, and Microbact 24E. The minimum inhibitory concentration (MIC) of selected antibiotics was determined by Vitek 2 compact system. Whole genome sequencing was conducted on eighteen isolates that met pre‐sequencing quality standards, utilizing the Illumina HiSeq platform. Sequence types and AMR genes were determined using publicly available tools. Interestingly, all isolates showed 100% phenotypic susceptibility to the tested antibiotics. Notably, several rare *Salmonella enterica* serovars were identified among the sequenced strains; Koketime (*n* = 2), Hadar (*n* = 3), Banalia|Tounouma (*n* = 10), Hermannswerder (*n* = 2), and Chomedey|Glostrup (*n* = 1). While most of the sequenced *Salmonella enterica* strains (15 out of 18) lacked AMR genes besides efflux transporter gene, a strain of Chomedey|Glostrup serovar exhibited genes associated with reduced susceptibility to aminoglycosides (*aph(3′)‐lb*, aph(6)‐Id), quinolones (*qnrB*), sulphonamides (*sul2*), and tetracycline (*tet(A)*), while Koketime strains possessed fosfomycin resistance genes (*fosA7*) besides the efflux genes. The absence of phenotypic and genotypic AMR in most of the isolates highlights the possibility that AMR could be controlled in livestocks in developing countries.

## Graphical abstract

This study examined *Salmonella enterica* isolated from cattle faeces on a Nigerian farm. All isolates exhibited complete susceptibility to tested antibiotics, and whole‐genome sequencing revealed minimal antimicrobial resistance gene carriage. These findings demonstrate a notably low resistance burden which is an uncommon observation in many low‐and middle‐ income African settings, underscoring the crucial role of prudent antibiotic use in livestock management.

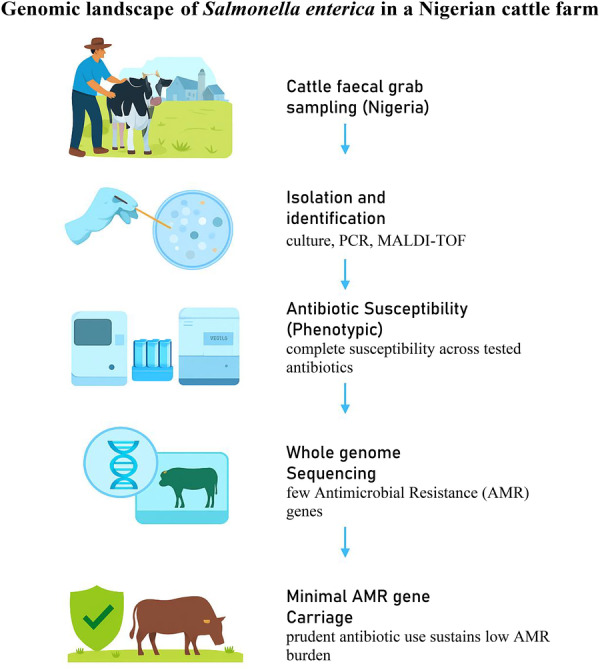

## 1 Introduction

Zoonoses are diseases that are naturally transmissible between animals and humans with or without vectors (Rahman et al. 2020). It is estimated that about 60% of all human infections are zoonotic while about 75% of all new human diseases over the last 10 years have been associated with either pathogens of animal origin or products from animal sources (Slingenbergh 2013). Salmonellosis—a disease caused by *Salmonella* spp., is adjudged the second most prevalent bacterial zoonotic disease, according to the most recent European Union One Health Zoonoses Report (Galán‐Relaño et al. 2023). It is a leading cause of bacterial food‐borne disease outbreaks in developed countries and a threat to public health in developing countries (Aarestrup et al. 2007; Andoh et al. 2017) particularly in Africa due to an already over‐burdened healthcare system. *Salmonella* consists of two species, *Salmonella enterica* and *Salmonella bongori. Salmonella enterica* consisting of more than 2600 serovars is by far the most implicated species in human and animal infections worldwide (Teklemariam et al. 2023; Hannah et al. 2023).

*Salmonella* infection contributes substantially to global morbidity and mortality, with over 93 million cases reported globally, resulting in 155,000 deaths annually (Majowicz et al. 2010; Ao et al. 2015; Eng et al. 2015; He et al. 2023; Lamichhane et al. 2024). In Africa, *Salmonella enterica* infection is suggested to be one of the most common causes of bloodstream infection (Breurec et al. 2019; Ikhimiukor et al. 2022). Bovine salmonellosis, on the other hand, has far‐reaching economic and health importance. Health‐wise, infected cattle may suffer weight loss, reduced milk, and meat yield and abortion in dairy cattle because of the debilitating effect of the disease (Peek et al. 2017). These result in lower productivity occasioned by the increased cost of treatment, and eventual mortality in cattle (Cummings et al. 2009; Asefa et al. 2023). The economic and medical importance of zoonotic salmonellosis is of global concern due to its shared importance in human and animal health (Chomel 2009).

Although salmonellosis is often reported as a foodborne disease, it has been established that about 10% of the cases are because of direct contact with animals shedding *Salmonella*.^3^ Shedding of *Salmonella* spp. has been widely documented in clinically healthy cattle, indicating that asymptomatic animals can serve as reservoirs and contribute to environmental contamination and zoonotic transmission (Nickodem et al. 2023; Bentum et al. 2025). Cattle faeces are important sources of *Salmonella* contamination and are considered a major route of transmission among cattle herd, food chain, and the environment (Burgess and Duffy 2011; Holschbach and Peek 2018). Cattle have also been reported to be naturally infected by *Salmonella* spp. (which implies that they can be infected through direct exposure to contaminated food, water, and the environment) with the prevalence of *Salmonella* isolation in bovine faeces ranging from about 0% to 62% (Elfenbein et al. 2013). It has been hypothesized that *Salmonella* is transmitted from the dam *in utero* to the fetus since faecal shedding has been reported in day‐old calves (Hanson et al. 2016). The prevalence of *Salmonella* carriage in cattle is reported to be highest in developing countries (Ejo et al. 2016). The global pooled *Salmonella* prevalence is about 9% with a range of 2%–16% in Europe and North America respectively, and the most diverse serotypes (74 out of 143 serotypes) are found in Africa (Gutema et al. 2019). The prevalence of *Salmonella* carriage in cattle is reported to be 10% in Nigeria (Umeh and Paulinus Enwuru 2014).

Since the first documented incidence of antibiotic resistance in *Salmonella* in the 1960s, there has been an upsurge in the prevalence of resistant *Salmonella* strains in both developed and developing countries (Eng et al. 2015; Gangathraprabhu et al. 2020; El‐Hanafi et al. 2023). Several studies have reported Multi‐Drug Resistant (MDR) serotypes possessing the ability to produce different hybrid plasmids (Lindsey et al. 2009; Bartsch et al. 2023; Egorova et al. 2023). These hybrid plasmids can carry multiple resistance genes and virulence factors, making infections more severe and difficult to treat, while also facilitating the spread of these traits across bacterial populations (McMillan et al. 2020). Livestock management is one of the prominent drivers of antimicrobial resistance (AMR) in zoonotic pathogens including *Salmonella* spp. due to the widespread incorporation of antibiotics in animal feed (Alhaji and Isola 2018). Global consumption of antibiotics in livestock production in 2010 was about 63,151 tons; it is projected to increase by about 67% by 2030 (Van Boeckel et al. 2015). For optimum productivity, farmers administer prophylactic doses of antibiotics to cattle herd particularly when stocking new calves to prevent diseases on their farm. Antibiotics are also administered to a herd of asymptomatic animals regarded as “at‐risk,” which were in close contact with animals showing symptoms of infectious disease (Paulson et al. 2015; de Mesquita Souza Saraiva et al. 2022). This practice referred to as metaphylaxis (mass medication to control disease in at‐risk animals) is thought to be a proven method for reducing morbidity and mortality in cattle, particularly against bovine respiratory disease (Horton et al. 2023). Subtherapeutic doses of antibiotics are added to feed and/or water over a period to enhance growth promotion and production efficiency by some commercial farmers (Canton et al. 2021).

Commonly administered classes of antibiotics in livestock management are tetracyclines, β‐lactams, quinolones, sulfonamides, macrolides, and glycopeptides with oral route of administration being the preferred route (Pijpers et al. 1991; Nielsen and Gyrd‐Hansen 1996; Broens and van Geijlswijk 2018). In Nigeria, tetracycline (33.6%), fluoroquinolones (26.5%), and aminoglycosides/beta‐lactams (20.4%) are the commonest antibiotics used in food animal production (Adesokan et al. 2015). Some of these antibiotics are not completely metabolized, the unabsorbed antibiotics can significantly alter the gut microbiota (Ramirez et al. 2020). A number of these antibiotics are excreted unchanged and remain active in faeces creating selective pressure on environmental bacteria, giving rise to the emergence of AMR. The emergence and dissemination of AMR pathogens have been associated with heightened morbidity and mortality rates across species, reduced farm productivity, and compromised food security (Van Boeckel et al. 2019; World Health Organization 2020). These impacts underscore the urgent need for comprehensive strategies to mitigate the spread of AMR, particularly in the context of agriculture and public health systems (Robinson 2018). There have also been reports of increasing food safety concerns due to the persistence of antibiotic residues in animal products with far‐reaching health implications (Van Boeckel et al. 2015).

More information is needed on comprehensive phenotypic and genetic AMR determinants in *Salmonella enterica*. isolated from cattle farms in some developing countries where AMR is endemic. This will provide more information on emerging AMR that could lead to global spread if not curtailed. In this cross‐sectional study, we selected one of the largest dairy cattle farms in Ibadan Nigeria, with single time point sampling to provide a snapshot of the prevalence of *Salmonella enterica* strains in cattle faeces and their AMR determinants.

## 2 Materials and Methods

### 2.1 Ethical Statement

All animal handling procedures were reviewed and approved by the Animal Care and Use Research Ethics Committee (ACUREC) of the University of Ibadan with the approval number UI‐ACUEC/17/0011.

### 2.2 The Study Cattle, and the Farm

The study site was the Teaching and Research Farm of the University of Ibadan (UI‐T& RF), Ibadan, Nigeria. It is an academic‐based research farm, that is responsible for meat and dairy supply to the community. The dairy cattle (Sokoto Gudali breed) used in this study were ear‐tagged, average age of 2.0 ± 0.5 years, born and raised on the research farm. All the animals sampled were confirmed to be healthy by the resident veterinarian. They do not come in close contact with other animals or livestock from the community. Existing farm hygiene and biosecurity include a fenced paddock with a gatehouse, proper drainage, clear zoning, and controlled access to the farm. New (occasionally for breeding programs) and sick cattle are usually quarantined, while regular vaccination and parasite control ensure cattle health. Clean housing, safe feed storage, and a clean water supply from a borehole within the farm are used to prevent contamination. Animal faeces are removed frequently. Also, protective gears are worn by farm staff, vehicle disinfection, manure management and pest control are put in place to prevent disease spread. The farm management is supervised by faculty members of the Department of Animal Science, while the herd healthcare including antibiotic stewardship is strictly coordinated by the University's Veterinary Teaching Hospital. The resident veterinarians ensure antimicrobial stewardship by administering antibiotics to animals only for therapeutic purposes. Antibiotics are only administered to clinically ill animals after diagnosis and antimicrobial susceptibility testing. Antibiotic feed additives are not used on the farm. Although detailed disinfection and antibiotic administration logs were not accessible for this study due to farm record keeping protocols, information provided by the resident veterinarian was considered reliable for describing antimicrobial usage practices.

**Figure 1:**
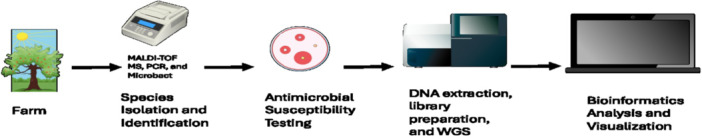
An overview of the study.

The flow diagram of the methodology used in this study is shown below (Figure 1).

### 2.3 Isolation of *Salmonella enterica*

*Salmonella enterica*. was aseptically cultured from a total of 138 non‐repetitive, fresh faecal samples collected directly from the rectum of 138 cattle, housed in paddocks over a period of 5 months at the University of Ibadan Dairy Farm. Each selected animal was restrained in a standing position, and faecal samples were collected using the faecal grab method, in which a gloved hand was gently inserted into the rectum of each animal to stimulate defecation and collect faecal samples. Only one sample was collected from each animal during the study period, A new pair of disposable gloves was used for each animal to prevent cross contamination. The samples were collected in pre‐labelled, sterile, wide‐mouth screw cap sample bottles, immediately stored in a cool box containing ice packs, and transported to the laboratory within 2 h of collection for *Salmonella enterica* isolation.

All cattle sampled were confirmed to be clinically healthy at the time of sampling, based on physical examination by a qualified veterinarian. They showed no signs of illness such as fever, anorexia, diarrhea, nasal discharge, abnormal behaviour and had normal appetite. According to the cattle handlers, there was no history of antibiotic treatment or disease within at least 4 weeks before sampling. This ensured that the animals represented an apparently healthy population for the purposes of this study. *Salmonella enterica* was isolated from cattle faeces by a modification of the method of International Standard Organization (ISO‐6579, 2000) as follows; 10 g of cattle faeces was enriched in 90 mL of buffered peptone water (Oxoid, UK) and incubated overnight (18‐20 h) at 37°C. One milliliter of the enriched cultured peptone water sample was transferred into 10 ml of Tetrathionate‐Novobiocin broth (Oxoid, UK) and incubated at 37°C for 24 h. A loopful of the broth culture was then inoculated on Xylose Lysine Deoxycholate agar (Oxoid, UK) and *Salmonella*‐*Shigella* agar (Oxoid, UK), incubated for 24 h at 37°C. On XLD, presumptive *Salmonella enterica* colonies appear as red colonies with black centres, indicating hydogen sulphide (H_2_S) production, while on SS agar they appear colourless or pale colonies with black centres due to their inability to ferment lactose and their production of H_2_S. Characteristic *Salmonella* colonies were further stabbed in Triple Sugar Iron agar with an inoculating wire and incubated for 24 h at 37°C. Colonies with typical *Salmonella* characteristics were selected for further identification.

### 2.4 Identification of *Salmonella enterica* by Matrix‐Assisted Laser Desorption/Ionization Time‐of‐Flight Mass Spectrometry (MALDI‐TOF MS)

All 68 isolates presumptively identified as *Salmonella enterica* based on growth on selective and differential media were further confirmed using MALDI‐TOF, a mass spectrometry identification method. Before identification, pure bacterial strains were subcultured on MacConkey agar plates for 24 h. Thin smears of pure isolated colonies to be identified were placed on the target MALDI plate, this was overlaid with 1 µl of saturated solution of α‐cyano‐4‐hydroxycinnamic acid in 50% acetonitrile and 2.5% trifluoroacetic acid (matrix solution), and then air dried at room temperature to allow co‐crystallization of the matrix‐sample (Akinbami et al. 2018) The MALDI plates were analysed in MALDI‐TOF (VITEK MS, Biomerieux, Nuertingen, Germany) and identification of the test organisms were achieved by a comparison of the mass spectra of the test isolate with reference spectra from the integrated database provided by the manufacturer. The similarity log‐score thresholds of were used for identification (Seng et al. 2010).

### 2.5 Identification of *Salmonella enterica* by Pcr

Out of the 68 isolates presumptively identified as *Salmonella enterica*, 32 were confirmed as *Salmonella enterica* by MALDI‐TOF MS. These 32 confirmed isolates were further subjected to PCR analysis‐a method to detect or confirm the presence of target genes. Three pure colonies of each of the 32 *Salmonella enterica* isolate previously identified by MALDI TOFs were suspended in 500 µL of molecular grade water, boiled at 100°C for 10 min, cooled on ice, and then centrifuged at 10,000 rpm for 10 s. The supernatant containing the DNA was removed and used as DNA template for PCR reaction targeting the 284 bp region of *Salmonella invA* gene with the primers: Sal 1 (5′‐GTGAAATTATCGCCACGTTCGGGCAA) and Sal 2 (5′‐ TCATCGCACCGTCAAAGGAACC) (Shanmugasamy et al. 2011), in a 25 µL reaction tube containing Ready‐To‐Go™ PCR master mix beads (GE Healthcare Lifescience illustra PuReTaq) with the isolate's DNA (1 µL) as the template, and, 0.5 µL each of the primer and 10.5 µL of nuclease free water. *S. enterica* serovar Typhimurium ATCC 14028 and molecular grade water were used as positive and negative controls respectively. The amplification was done in an Eppendorf Thermocycler (Applied Biosystem, Singapore) with PCR conditions consisting of an initial incubation step at 94°C for 1 min, 35 cycles of 94°C for 1 min, followed by annealing at 64°C for 30 s and elongation at 72°C for 30 s, followed by 7 min at 72°C. The amplicons were visualized under UV light in a 1% agarose w/v gel with an expected amplified PCR product of 284 bp (Shanmugasamy et al. 2011).

### 2.6 Identification of *Salmonella enterica* by Microbact 24E System

Thirty‐two identified *Salmonella enterica* isolates from MALDI‐TOF and PCR were tested with Microbact 24E system (Oxoid, UK) and the results interpreted according to the manufacturer's instructions. The Microbact 24E system (Oxoid Ltd., UK) is a standardized biochemical testing method used for the identification of Enterobacteriaceae and other Gram‐negative bacteria. The system employs a panel of 24 miniaturized biochemical tests that evaluate metabolic and enzymatic characteristics of bacterial isolates, enabling rapid identification. After incubation, color changes in the wells were recorded according to the reference chart, indicating positive or negative results for each test. The biochemical reaction pattern was analyzed using the Microbact Computer‐Aided Identification Package, which compares the observed pattern against a database to determine the most probable bacterial species or genus (Mailafia et al. 2017).

### 2.7 Determination of Antimicrobial Susceptibility of Identified *Salmonella enterica*

The antimicrobial susceptibility of the 32 *Salmonella* isolates was determined by VITEK2 compact system (AST‐N214 cards, Biomerieux, Nuertingen, Germany). The card was chosen because the antibiotics in it reflects the antibiotics generally used in the studied community (Nigeria). The antibiotics panel consists of ampicillin, ampicillin‐sulbactam, tetracycline, gentamicin, trimethoprim‐sulfamethoxazole, cefotaxime, imipenem, meropenem, tigecycline, cefuroxime, ciprofloxacin, piperacillin/tazobactam, ertapenem, ceftazidime, moxifloxacin and cefpodoxime according to the manufacturer's instructions. The *Salmonella* isolates were classified as susceptible (S), intermediate (I) or resistant (R) by the automated machine.

### 2.8 DNA Extraction, Library Preparation, and Whole Genome Sequencing

The isolates genomic DNA was extracted using the Wizard DNA extraction kit (Promega; Wisconsin, USA) following the manufacturer's guidelines. DNA purity was assessed using a NanoDrop spectrophotometer (Thermo Scientific, USA) by measuring the A260/A280 ratio while quantification of the extracted DNA was performed on a Qubit fluorometer (Invitrogen; California, USA) using the dsDNA Broad Range quantification assay. Out of the 32 *Salmonella* isolates, only 18 met the stringent quality control criteria for purity (A260/A280 between 1.8 and 2.0), DNA integrity (as confirmed by agarose gel electrophoresis), and concentration, ensuring suitability for downstream library preparation and sequencing. Subsequently, double‐stranded DNA libraries were prepared by fragmenting the extracted DNA, tagged with adapters, and prepared for sequencing using NEBNext Ultra II FS DNA library kit for Illumina featuring 384‐unique indexes (New England Biolabs, Massachusetts, USA; Cat. No: E6617L). The sequencing process was conducted on an Illumina HiSeq X10 (Illumina, California, USA) platform at the Wellcome Sanger Institute.

### 2.9 Bioinformatics Analyses

Computational tools were used to process, analyze, and graphically present genomic data for interpretation. The sequence reads obtained from Illumina runs underwent *de novo* assembly, following the Global Health Research Unit (GHRU) protocols (Underwood 2020). This process utilized a Nextflow workflow that encompassed various steps, including adapter trimming (trimmomatic v0.38), contamination detection (ConFindr v0.7.2), assembly (SPAdes v3.12.0), Quality Control (multiqc v1.7, qualifyr v1.4.4), and Bactinspector (v0.1.3). Subsequently, the *Salmonella* genome assemblies were analyzed using the *Salmonella* In‐Silico Typing Resource or the prediction of serovars and serogroups (Yoshida et al. 2016). Sequence types were determined using the Ariba software to determine the 7‐locus Achtmann‐scheme‐based MLST type using the profile and alleles found in the Pubmlst database (Jolley et al. 2004; Jolley and Maiden 2010). Additionally, determinants related to AMR, virulence, and plasmid replicons were identified following the GHRU protocols (Underwood 2020). Genomes were annotated using Bakta (v1.9.4) (Schwengers et al. 2021). To generate a mapping‐based phylogenetic tree, the *Salmonella* strain NZ_LR134158.1 (*Salmonella enterica* subsp. enterica serovar Goldcoast; https://www.ncbi.nlm.nih.gov/datasets/genome/GCF_900635695.1/) was selected by Bactinspector (https://gitlab.com/antunderwood/bactinspector) as the best reference. The minimum likelihood phylogenetic tree was constructed using IQtree implemented in the SNP nextflow pipeline. Isolate genomes were deposited in the European Nucleotide Archive (ENA) under project ID PRJEB8667 (https://www.ebi.ac.uk/ena/browser/view/PRJEB8667).

### 2.10 Visualization

Phylogenetic tree and metadata files were uploaded onto the Microreact platform for visualization. The Microreact link can be accessed here (https://microreact.org/project/2QVaRLUdRpgM1EsQDisJD3-wale-salmonella).

## 3 Results

### 3.1 Identification of *Salmonella enterica* Isolates

The prevalence of *Salmonella enterica* was determined in healthy cattle faeces in this study. Initially, 68 isolates from 138 different cattle faecal samples were presumptively identified as *Salmonella*. Of which, only 32 isolates (47.1%) were confirmed to be *Salmonella enterica* by MALDI‐TOF, PCR amplification and Microbact 24E, thereby giving 47.1% accuracy of cultural methods. Microbact 24E classified the isolates as three *Salmonella* Sub sp 5, one *Salmonella* Sub sp 3b and 28 *Salmonella* Sub sp 1 (Table 1). The prevalence of *Salmonella enterica* in cattle faeces was 23.2% on the studied farm.

**Table 1:** Identification of *Salmonella enterica* isolates with Microbact 24E.

S/N	Sample code	Microbact ref code	Microorganisms Identity	Probability
1	S1	77020621	*Salmonella* Sub sp 1	49.44%
2	S2	77420661	*Salmonella* Sub sp 3b	80.28%
3	S3	77020621	*Salmonella* Sub sp 1	49.44%
4	S4	77420621	*Salmonella* Sub sp 5	71.88%
5	S5	77010661	*Salmonella* Sub sp 1	72.57%
6	S6	77420621	*Salmonella* Sub sp 5	71.88%
7	S7	77021621	*Salmonella* Sub sp 1	98.83%
8	S8	77420621	*Salmonella* Sub sp 5	71.88%
9	S9	77020621	*Salmonella* Sub sp 1	49.44%
10	S10	77020621	*Salmonella* Sub sp 1	49.44%
11	S11	77020621	*Salmonella* Sub sp 1	49.44%
12	S12	77021621	*Salmonella* Sub sp 1	98.83%
13	S13	77020621	*Salmonella* Sub sp 1	49.44%
14	S14	77020621	*Salmonella* Sub sp 1	98.83%
15	S15	77020621	*Salmonella* Sub sp 1	49.44%
16	S16	67020621	*Salmonella* Sub sp 1	97.77%
17	S17	77020621	*Salmonella* Sub sp 1	49.44%
18	S18	77020661	*Salmonella* Sub sp 1	54.22%
19	S44	77220621	*Salmonella* Sub sp 1	74.19%
20	S47	77020621	*Salmonella* Sub sp 1	49.44%
21	S48	77020621	*Salmonella* Sub sp 1	49.44%
22	S49	77020621	*Salmonella* Sub sp 1	49.44%
23	S54	77020621	*Salmonella* Sub sp 1	49.44%
24	S56	77420621	*Salmonella* Sub sp 1	74.19%
25	S57	77020621	*Salmonella* Sub sp 1	49.44%
26	S58	77020621	*Salmonella* Sub sp 1	49.44%
27	S60	77000720	*Salmonella* Sub sp 1	49.65%
28	S62	77020621	*Salmonella* Sub sp 1	49.44%
29	S68	77020621	*Salmonella* Sub sp 1	49.44%
30	S70	77020621	*Salmonella* Sub sp 1	49.44%
31	S76	77020621	*Salmonella* Sub sp 1	49.44%
32	S77	77020621	*Salmonella* Sub sp 1	49.44%

### 3.2 Antibiotic Susceptibility Profile of the Tested *Salmonella enterica* Isolates

All the isolates were susceptible to the entire antibiotic panel tested using the Vitek 2 compact system. The panel consist of β‐lactams such as ampicillin, ampicillin‐sulbactam and piperacillin/tazobactam; Cephalosporins including cefuroxime, cefotaxime, ceftazidime, and cefpodoxime; carbapenems comprising imipenem, meropenem, and ertapenem. Also included were the aminoglycosides gentamicin, the Fluoroquinolone ciprofloxacin and moxifloxacin, as well as teteracycline, tigecycline and trimethoprim‐sulfamethoxazole.

### 3.3 *Salmonella enterica* Isolates Sequence Analysis

**Figure 2:**
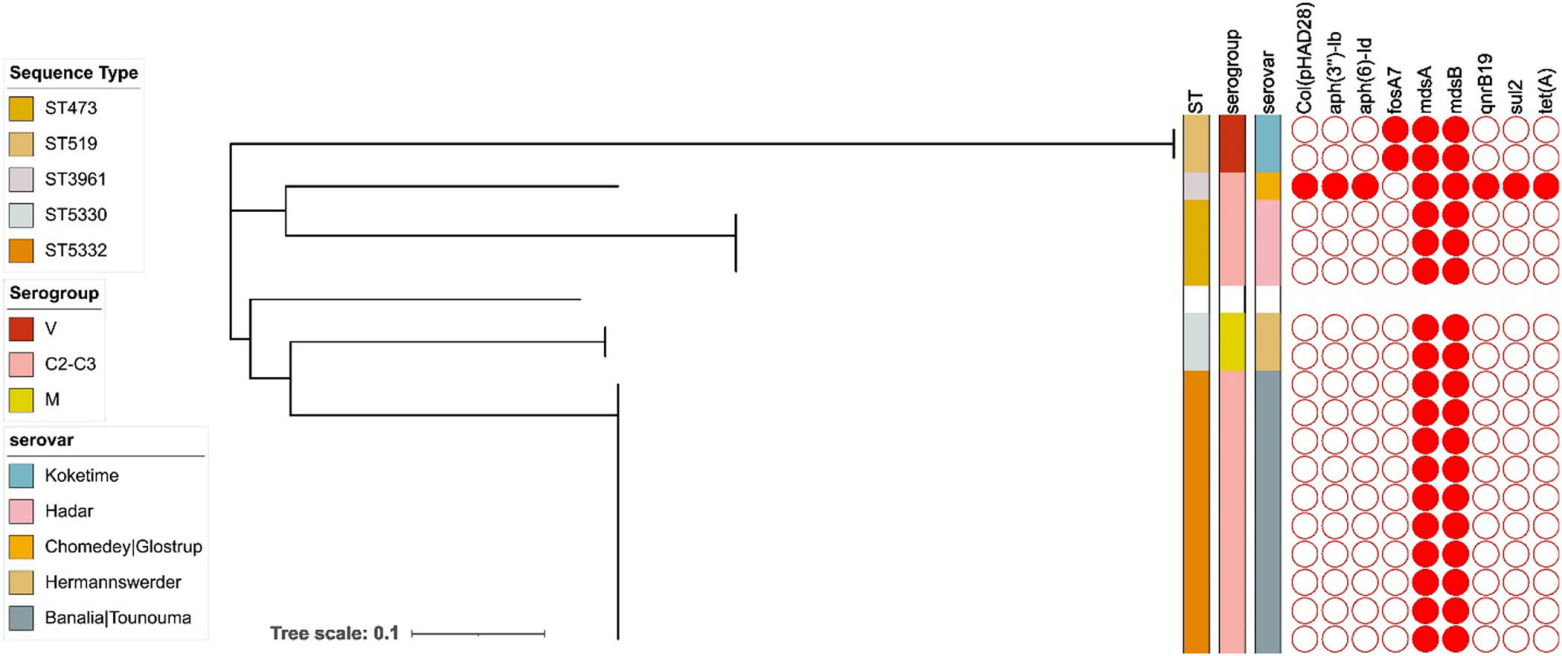
The sequence type of the isolated *Salmonella enterica* strains.

Eighteen of the identified *Salmonella enterica* strains that met pre‐sequencing quality standards were whole genome sequenced. These strains (genome length between 4.6 and 4.7 Mb) were classified into five Sequence Types (5332, 5330, 3961, 519, 473), corresponding to five distinct serovars (Banalia|Tounouma, Hermannswerder, Chomedey|Glostrup, Koketime, and Hadar). Each unique sequence type was consistently associated with a specific serovar, and these associations were reflected in the clustering observed in the phylogenetic tree based on both serovar and sequence type (Figure 2). Interestingly, 83% (15) of the sequenced *Salmonella enterica* isolates have no AMR genes besides efflux transporter gene, *Salmonella enterica* strains within sequence types 5332 (serovar Banalia|Tounouma; *n* = 10), 5330 (serovar Hermannswerder; *n* = 2), and 473 (serovar Hadar; *n* = 3) exhibited an absence of AMR genes besides efflux genes (Figure 2). In contrast, the solitary *Salmonella enterica* strain in sequence type 3961 (serovar Chomedey|Glostrup) harbored genes associated with resistance or reduced susceptibility to various antibiotics, including aminoglycosides (*aph(6)‐Id* and *aph(3′)‐Id*), fluoroquinolones (*qnrB19*), sulfonamides (*sul2*), and tetracycline (*tet(A)*). Interestingly, all except the *qnrB19* gene were carried on the same contig. However, the contig carrying the qnrB19 gene did not carry any other gene associated with AMR. Additionally, this strain carried the Col(pHAD28) plasmid. *Salmonella* strains affiliated with ST519 (serovar Koketime; *n* = 2) possessed only one gene conferring resistance to fosfomycin (*fosA7*), without the presence of any supplementary plasmids (Figure 2). Contigs carrying this gene (*fosA7*) carried the biofilm peroxide resistance gene (*bsmA*) and genes involved in conjugation (*traI*) and motility (*fimA*, *fimB*, *fimC*).

## 4 Discussion

The emergence and dissemination of AMR among zoonotic pathogens, particularly *Salmonella enterica*., pose significant challenges to global public health, especially in low‐ and middle‐income countries (Prestinaci et al. 2015). Several published studies agree that inappropriate use of antimicrobials among other factors contribute to emergence and spread of AMR in zoonotic pathogens (Aarestrup 2012; Adetoye et al. 2018; Hosain 2020; Caneschi et al. 2023; Ardakani et al. 2023; Olaru et al. 2023). This study focused on elucidating the prevalence, genetic diversity, and antibiotic susceptibility profiles of *Salmonella enterica*. isolated from healthy cattle faeces in a Nigerian University dairy farm where according to farm policy, antibiotics are administered only when clinically indicated by the resident veterinarian. The findings on genetic diversity of *Salmonella enterica* and no observed occurrence of phenotypic resistance to tested antibiotics in the current study reveal a nuanced scenario, emphasizing the potential benefits of careful antibiotic stewardship in agriculture to curb AMR while also highlighting the added value of genomic surveillance alongside conventional cultural methods.

Cultural methods alone yielded relatively low accuracy (47.1% confirmation rate), emphasizing the importance of confirmatory techniques like MALDI‐TOF and PCR for reliable pathogen identification (Patel 2015; Elbehiry et al. 2022; Ownagh et al. 2023). The detection of *Salmonella* spp. in 23.2% of the bovine faecal samples highlights the potential role of cattle as reservoirs for *Salmonella* transmission along the food chain (Topalcengiz et al. 2020; EFSA Panel on Biological Hazards et al. 2021; Bentum et al. 2025). Although direct comparisons with previous Nigerian studies are limited by difference in sampling location, farm management practices and methodologies, the observed prevalence underscores the ongoing need for local surveillance and control measures. Variation in breeding conditions, stocking density and hygiene practices can significantly influence both the prevalence of *Salmonella enterica* and the distribution of resistance genes. Poor farm environmental sanitation, as reported in other low and middle ‐income settings, may also contribute to the maintenance and circulation of *Salmonella enterica* in farm environments (Awad and Ghareeb 2014; Mkangara 2023).

Whole genome sequencing provided insights into the genetic diversity and population structure of the *Salmonella enterica* strains. Several rare *Salmonella* serovars were detected, which have been infrequently documented in prior studies or surveillance reports from Nigeria or the broader African region, including serovars Hadar, Koketime (Monte et al. 2021; Aworh et al. 2024). The identification of distinct sequence types associated with specific serovars underscores the genetic heterogeneity within the *Salmonella* population. For instance, sequence type ST5332 was consistently linked to the Banalia|Tounouma serovar. This genetic association underscores the potential for targeted interventions based on genomic signatures. Moreover, the association between sequence types and serovars elucidates potential evolutionary relationships and transmission dynamics among *Salmonella* strains circulating in the farm environment. Although there have been previous suggestions to phase out the use of serovar designations due to the polyphyletic nature of serovars and the error rates associated with serotyping, these designations remain a key method for typing *Salmonella* strains in microbiology (Alikhan et al. 2018). In silico serotyping tools still rely on MLST‐based predictions as a tiebreaker when genomic data lacks sufficient resolution. This highlights that the strong correlation between serotypes and sequence types observed in this study is not unexpected. Instead, it has been recommended that the medical community adopt a reporting format that uses the sequence type (ST) followed by the serovar (Chattaway et al. 2021). Notably, a few of the *Salmonella enterica* serovars observed in this study have been linked to significant foodborne outbreaks worldwide*. Salmonella* Hadar has been associated with several outbreaks linked to poultry products in Europe and North America (Brandenburg et al. 2024).

All isolates in this study were phenotypically susceptible to the entire antimicrobial panel tested with Vitek 2 compact system, which included antibiotics used in veterinary medicine (ampicillin, ampicillin–sulbactam, cefuroxime, tetracycline, gentamicin, and trimethoprim–sulfamethoxazole) and those critical for human healthcare such as carbapenems, tigecycline, moxifloxacin, and piperacillin/tazobactam. This observation of low detection of phenotypic AMR appears to contrast with global reports of increasing *Salmonella enterica* AMR (Talukder et al. 2023; Wang et al. 2025; Nazir et al. 2025). In a recent study, 81.8% of Salmonella strains isolated from cattle in Ethopia were reported to be multi‐drug resistant (Seid et al. 2025), this is in tandem with a Nigerian study where multi‐drug resistance was observed in 81.5% of *Salmonella enterica* strains isolated from cattle, cattle handlers and abattoir environment (Aworh et al. 2024). Casaux et al. (2019) have equally reported high levels of AMR among 41 *Salmonella* isolates from dairy cattle in Uruguay, where 92.7% showed resistance to tetracycline and 34% classified as multidrug‐resistant (MDR) with resistance to up to six antibiotics, this showed a stark contrast with our study, where all *Salmonella enterica* isolates were susceptible to the antibiotics tested.

The absence of detectable phenotypic expression of antibiotic resistance among the strains investigated may reflect the farm's prudent use of antibiotics (EMA and EFSA European Medicines Agency and European Food Safety Authority 2017; Jayarao et al. 2019). However, detailed farm records of antimicrobial use, disinfection practice, and AST protocols were not accessible due to farm record keeping protocol, we cannot definitively link the susceptibility pattern observed to specific farm management interventions.

Interestingly, whole‐genome sequencing revealed that most of the isolates lack detectable AMR genetic determinants altogether except efflux transporter gene The genotypic results also correlate with the phenotypic results for 83% of the *Salmonella enterica* isolates in this study. This absence of genotypic AMR determinants could be due to minimal antimicrobial selective pressure on the farm, given the restricted therapeutic‐only use of antibiotics, as well as the potential dominance of naturally susceptible *Salmonella* lineages in this cattle population. In addition, limited opportunities for horizontal gene transfer, possibly due to biosecurity measures and herd isolation from external livestock. However, there is presence of certain AMR genes in some isolates (17%). A single strain (ST3961) out of the 18 tested strains harboured *tet(A)*, *sul2*, *qnrB19*, and *aph* genes, while two ST519 isolates carried *fosA7* This multidrug resistant strain, identified as the Chomedey|Glostrup strain (ST3961), carried genes associated with reduced susceptibility to aminoglycosides, fluoroquinolones, sulfonamides, and tetracycline. This aligns with earlier findings that *Salmonella enterica* serovars can carry multiple resistance genes, posing a risk to both human and animal health (Nair et al. 2018; Moraes et al. 2024) The presence of resistance genes without corresponding phenotypic resistance could possibly be attributed to low levels expression of the gene under laboratory conditions, the efflux genes detected might not confer strong enough resistance to be detected by standard susceptibility tests and some resistance mechanisms may be nonfunctional due to mutations or incomplete integration of the genes. This phenomenon has been well documented in previous studies, where *Salmonella enterica* isolates harbored AMR determinants despite exhibiting phenotypic susceptibility during routine antimicrobial susceptibility testing (Bolkenov et al. 2024), these “silent” resistant genes can be transferred horizontally between bacteria even without phenotypic resistance expression. The presence of the *tetA* gene, despite no phenotypic resistance to tetracycline observed in AST results, may be due to several factors. These include the possibility of gene inactivation, insufficient expression levels, or the absence of necessary regulatory elements to confer resistance under the testing conditions (Yee et al. 2021). Similarly, isolates of ST519 harbored FosA7, conferring fosfomycin resistance, which was not tested phenotypically as fosfomycin was not part of the AST panel.

From a One Health perspective, the cattle's environment such as soil, water sources and residual cattle faeces could also serve as reservoirs of AMR genes, posing a potential public health risk if these determinants are transferred to pathogenic strains under antibiotic selection pressure (Koutsoumanis et al. 2021) Additionally, the presence of resistance genes in this specific serovar, despite overall phenotypic antibiotic susceptibility, emphasizes the complex interplay between genetics and antibiotic exposure and the need for a multifaceted approach to AMR surveillance. These findings underscore the importance of integrating genomic data with phenotypic results as genomic surveillance complements phenotypic testing by detecting “silent” AMR genes that might otherwise go unnoticed, providing a more robust understanding of the AMR landscape in livestock farming (Deekshit and Srikumar 2022).

The co‐occurrence of the Col(pHAD28) plasmid with the quinolone resistance gene (*qnrB19*) in the Chomedey|Glostrup (ST3961) strain was observed in a previous study examining *Salmonella* strains in the environment, suggesting that horizontal gene transfer could facilitate the dissemination of resistance determinants among different bacterial populations (Jibril et al. 2021). Furthermore, the detection of fosfomycin resistance genes in *Salmonella* Koketime strains highlights the importance of monitoring emerging resistance mechanisms, even in antimicrobial classes less commonly used in veterinary medicine. The presence of aminoglycoside resistance genes aph(6)‐Id and aph(3’)‐Id, fluoroquinolone resistance gene qnrB19, sulfonamide resistance gene sul2, and tetracycline resistance gene tet(A) in a phenotypically susceptible isolate is of particular concern because these antimicrobial classes include agents that are recommended in various settings for the treatment of important human infections such as urinary tract infections, gastrointestinal infections, and certain systemic bacterial diseases (Kim and Hooper 2014; Salam et al. 2023). These findings are consistent with past research showing that resistance to less frequently used antibiotics can still occur and spread among bacterial strains (Andersson and Hughes 2011).

The study's single‐site, cross‐sectional design was intended to provide a proof‐of ‐concept data on *Salmonela enterica* genomic epidermiology. Future research should incorporate multiple farms representing varied management systems, comprehensive farm antimicrobial usage records and environmental samples to better capture AMR transmission pathways. The tested antibiotics covers the range of antibiotics that are usually use on farms and the country of study, however, the breadth of the antibiotic panel used may not have been comprehensive enough to detect all potential phenotypic resistance. For example, resistance genes for fosfomycin were identified in one strain, but it was not part of the panel tested. The inclusion of broader AST panels in future tests would improve genotype‐phenotype correlation and strenghten risk assessment. We also observed that some *Salmonella enterica* genomes exhibited dual serovar identities (e.g.,“Banalia|Tounouma”). This ambiguity arises from limitations in the SISTR platform, which relies on core genome MLST for serovar prediction. In these cases, only 71‐75 cgMLST loci matched the closest reference genomes, reducing the confidence in distinguishing between closely related serovars. This underscores the importance of complementing genotypic predictions with traditional serotyping methods to ensure accurate serovar identification when genomic resolution is insufficient. Moreover, there is a need for future research to embrace a One‐Health surveillance approach. This approach would facilitate the investigation of the genomic relatedness of *Salmonella enterica* across various sampling matrices and enable the identification of AMR determinants contributing to the development and dissemination of antibiotic resistance. By adopting a holistic perspective that encompasses human, animal, and environmental health, One‐Health surveillance studies can offer valuable insights into the complex dynamics of AMR and inform strategies for its mitigation.

## 5 Conclusion

This study report distribution and low detection of phenotypic and genotypic AMR resistance in *Salmonella enterica* strains isolated from cattle feces in a cattle farm in Nigeria under an antibiotic stewardship regime, We also report few isolates without phenotypic resistance to the tested antibiotics have genotypic evidence of AMR determinants. These findings emphasize the possibility of having low AMR occurrence in world regions with high endemic AMR prevalence and that AMR surveillance should integrate phenotypic testing with genomic analysis to capture both expressed and “silent” resistance to enable proactive interventions to preserve antimicrobial efficacy in veterinary and human medicine.

## Author Contributions

**Adewale A. Adetoye:** methodology, investigation, writing – original draft. **Ayorinde O. Afolayan:** data curation, investigation, formal analysis, visualization, writing – original draft. **Olabisi C. Akinlabi:** data curation, writing – review and editing. **Stella E. Ekpo:** data curation. **Isaac O. Olatoye:** methodology, supervision, writing – review and editing. **Funmilola A. Ayeni:** conceptualization, supervision, project administration, writing – review and editing.

## Conflicts of Interest

The authors declare no conflicts of interest.

## Data Availability

The data that support the findings of this study are openly available in European Nucleotide Archive (ENA) at https://www.ebi.ac.uk/ena/browser/view/PRJEB8667, reference number PRJEB8667.
